# Postsurgical Adhesions: Is There Any Prophylactic Strategy Really Working?

**DOI:** 10.3390/jcm12123931

**Published:** 2023-06-08

**Authors:** Irina-Maria Flutur, Dan Nicolae Păduraru, Alexandra Bolocan, Alexandru Cosmin Palcău, Daniel Ion, Octavian Andronic

**Affiliations:** 1 Carol Davila University of Medicine and Pharmacy, 050474 Bucharest, Romania; irina.flutur@stud.umfcd.ro; 2 Department of General Surgery, Carol Davila University of Medicine and Pharmacy, 050474 Bucharest, Romania; alexandra.bolocan@umfcd.ro (A.B.); daniel.ion@umfcd.ro (D.I.); octavian.andronic@umfcd.ro (O.A.); 3 IIIrd Clinic of General and Emergency Surgery, University Emergency Hospital of Bucharest, 050098 Bucharest, Romania; alexandru-cosmin.palcau@drd.umfcd.ro

**Keywords:** postoperative adhesions, adhesion barriers, prophylaxis, prevention

## Abstract

Postoperative adhesions are a frequent complication encountered after surgical procedures, mainly after intraperitoneal interventions. To this day, the pathophysiological mechanism behind the process of adhesions formation is not completely known. There are many strategies proposed as prophylaxis methods, involving surgical techniques, drugs or materials that prevent adhesions and even state of the art technologies such as nanoparticles or gene therapy. The aim of our review is to present these innovative approaches and techniques for postoperative adhesions prevention. After a thorough scientific database query, we selected 84 articles published in the past 15 years that were relevant to our topic. Despite all the recent groundbreaking discoveries, we are at an early stage of understanding the complexity of the adhesion formation mechanism. Further investigations should be made in order to create an ideal product for safe clinical use for prevention.

## 1. Introduction

Postsurgical adhesions are a common complication after surgical procedures, especially after interventions on the abdomen and pelvis. They can cause chronic pain, intestinal obstruction and fertility issues in women, plus they can also make future surgery more difficult [1]. Postoperative adhesions represent a relevant problem for the healthcare system and a satisfactory solution to it is yet to be found [2].

The pathophysiology of adhesion formation is still incompletely known, although some progress has been achieved in this regard in past years. Many prevention strategies have been proposed, such as modification of the surgical technique, using drugs that exert pharmacological effects and interfere with the pathophysiologic mechanism of adhesions or drugs that do not possess such properties and even gene therapy. A lot of new products have already proved their efficacy in studies on laboratory animals, but none of them have yet been approved for clinical use [3].

The purpose of this review is to present the postsurgical adhesions prevention methods that have been tested thus far. The mechanism of action and experimental efficacy will be analyzed for each prophylaxis measure.

## 2. Materials and Methods

The databases Pubmed, Scopus and Web of Science have been searched using a query that incorporated relevant keywords. The search query included “(postoperative adhesions OR postsurgical adhesions)” AND “(prevent* OR prophylaxis*)”. These terms were chosen based on their relevance to our topic and their likelihood of providing the most applicable articles for our review.

In our study we only included free full-text articles published in the past 15 years to ensure the quality and relevance of our research. However, this time limit is flexible and serves as a general guide in our search process.

After conducting the search and evaluating according to the relevance of the article to the topic, the quality of the research and the reliability of the data presented, 84 articles have been included. The articles selected for our final analysis provide a comprehensive overview of the current state of knowledge on the prevention and prophylaxis of postoperative adhesions.

## 3. Results and Discussion

In order to understand how the products mentioned in this review act to prevent adhesions, one must be acquainted with the structure of the peritoneum and the pathophysiology of adhesion formation.

1. The structure of the peritoneum

The peritoneum is a delicate membrane consisting of a layer of mesothelial cells which is supported by connective tissue. It covers the abdominal organs and protects them from friction and infection, but it also synthetizes molecules that participate in the inflammatory and healing process [4].

2. Pathophysiology of adhesion formation

As mentioned before, the pathophysiology of adhesion formation is still incompletely known. However, some of the mechanisms have been distinguished, as follows:

1. The lysis of fibrin and extracellular matrix is inhibited.
2. An inflammatory process arises, which leads to cytokines and TGFβ production; TGFβ is also involved in fibrosis regulation.
3. Mesothelial cells and submesothelial fibroblasts are affected by the lack of oxygen caused by blood flow interruption; consequently, hypoxia inducible factor 1α (HIF-1α) and vascular endothelial growth factor (VEGF) expression is stimulated; the result of this is collagen synthesis and angiogenesis [5].

The order of events is the following:

➢ The peritoneum is injured during surgery.
➢ Blood vessels become more permeable and this gives rise to inflammatory exudate.
➢ During the inflammatory response different types of cells are activated, such as mast cells, neutrophils, plasma cells and monocytes, while fibrin is deposited at the injury site.
➢ Normally, fibrin should become degraded by plasmin, but tissue hypoxia interferes with this process.
➢ Fibroblasts infiltrate the fibrinous mass and an adhesion will develop at the site [2].

The probability that fibrinolysis does not occur and that an adhesion forms is determined during the first 3 to 5 days after the surgical intervention. Therefore, this period of time is the key moment for prophylaxis [6].

3. Prevention of postsurgical adhesions

### 3.1. Surgical Technique

It is acknowledged that the surgical technique is one of the factors involved in the occurrence and extent of postoperative adhesions. For this reason, it is recommended that the tissues are handled gently. Electrocautery use, duration of surgery, pneumoperitoneum pressure, tissue dehydration and foreign body contamination should be reduced as much as possible [2].

Some studies claim that laparoscopy generates fewer adhesions in comparison with laparotomy [7,8,9], but there are also studies where no difference has been noticed between the two procedures [10]. Laparoscopy is less traumatizing to tissues, but it involves pneumoperitoneum establishment, which offers surgeons better surgical field visibility and space to handle the instruments. Carbon dioxide has been chosen for this purpose because of its affordable price, high water solubility and non-flammable property. After surgery, carbon dioxide is absorbed into the circulatory system and then eliminated through the lungs [4].

Animal testing has revealed that carbon dioxide stimulates adhesion formation through tissue dehydration, induction of a certain degree of cell hypoxia and pH decrease. These effects are proportional to pneumoperitoneum duration and gas insufflation pressure [11].

During a randomized controlled trial performed on endometriosis patients, the experimental group received a mixture of carbon dioxide, nitrous oxide and oxygen as insufflation gas, which was humidified using a heparin solution. Moreover, the peritoneal cavity was cooled and intramuscular dexamethasone was injected at the end of the intervention. The control group was only administered carbon dioxide at 37 °C intraperitoneally. While all the women in the control group developed severe postsurgical adhesions, just one quarter of the experimental group members each acquired a small adhesion [12].

On that account, modifying the parameters of the gas insufflated in the abdominal cavity is necessary in order to reduce the adhesion incidence after surgery.

### 3.2. Drugs That Exert Pharmacological Effects

Numerous studies have been carried out regarding drugs with anti-adhesive effect after abdominal surgery. A summary of them can be found in Table 1.

➢ Drugs that interfere with the renin-angiotensin-aldosterone system (RAAS):

Angiotensin II is involved in adhesion formation. It acts by binding to angiotensin II type 1 receptors (AT1), thus stimulating inflammation, fibrosis and extracellular matrix deposition [13]. Using AT1 receptor blockers on laboratory rodents has reduced adhesion generation [14].

The effect of angiotensin on type 2 receptors (AT2) is opposed to the one on AT1, since it inhibits fibrosis. Accordingly, Compound 21 (C21), which is an AT2 agonist, has decreased intraperitoneal TGFβ levels in animal subjects and has successfully prevented abdominal adhesion formation [15].

Angiotensin-converting enzyme (ACE) inhibitors are another available option for interference with RAAS. Lisinopril has suppressed TGFβ expression and has proved to be an efficient anti-adhesion drug in animal experiments [16].

➢ Hypoxia-Inducible Factor (HIF) and N-acetylcysteine (NAC)

Since hypoxia is involved in the process of adhesion formation, a small molecule HIF inhibitor called YC-1 has been tested on mice (its chemical formula is 3-(5′-hydroxymethyl-2′-furyl)-1-benzyl indazole). It was concluded that HIF inhibition stimulates fibrinolysis and blocks angiogenesis, as well as fibroblast and macrophage activation. Through these mechanisms, adhesion production is impaired [17].

During hypoxic states, reactive oxygen species are created. As a way to counteract their effects, N-acetylcysteine (NAC), an antioxidant, has been used on laboratory animals. The results were satisfactory, since anti-inflammatory and fibrinolytic properties were noticed [18,19,20].

➢ 3-hydroxy-3-methylglutaryl coenzyme A (HMG-CoA) reductase inhibitors

Statins are widely used hypolipidemic drugs which act through HMG-CoA inhibition. In addition, they reduce the levels of cytokines associated with fibrosis (for example TGFβ), raise tissue plasminogen activator (tPA) expression and decrease plasminogen activator inhibitor (PAI-1) expression. After the confirmation of their beneficial effect on rodents, two retrospective cohort studies were analyzed, so that a link between statin administration and adhesion prevention in humans could be discovered. By studying patients who had surgery in the UK between 1996 and 2013 and others who had surgery in the USA between 2000 and 2016, a comparison was made between those who were already under treatment with statins at the time of the intervention and those who were not. Patients treated with this drug class had fewer complications caused by adhesions (such as intestinal occlusion or adhesiolysis requirements) [21].

➢ Neurokinin-1 receptor (NK-1R) antagonists

Substance P binds to the NK-1R, thus amplifying the fibrotic process. Intraperitoneal injection of a NK-1R antagonist (CJ-12,255, Pfizer) in laboratory rats has reduced adhesions incidence by half and has also enhanced the activity of matrix metalloproteinases in the peritoneal fluid [22]. A similar trial has also revealed fibrinolysis stimulation and oxidative stress reduction [23].

➢ Lubricin

Lubricin is a glycoprotein, which is usually found on the surface of articular cartilage and serves as a lubricant and an anti-adhesive agent. Although effective against adhesion formation during animal testing, it seems that lubricin delays the healing of surgical wounds [24].

➢ Chymase inhibitors and sodium cromoglycate

Chymase is one of the inflammatory mediators released from mast cells, which in turn participate in adhesion production. Chymase activates TGFβ1 and stimulates fibrosis. Its inhibitor, Suc-Val-Pro-Pheᴾ-(OPh)₂, has been successfully used for cardiac [25] and peritoneal [26] adhesion prophylaxis in hamsters. TY-51184 was also proved efficient as an intraperitoneal injection [27], as was NK3201 during oral administration [28].

Sodium cromoglycate hinders the release of mediators (such as histamine) from mast cell granules. In rabbits, cromoglycate inhibits adhesion development, while adding aprotinin and dexamethasone to it which potentiates that effect [29].

➢ Anti-inflammatory drugs

Nonsteroidal anti-inflammatory drugs (NSAIDs) work by inhibiting the enzyme cyclooxygenase (COX), which diminishes the number of prostaglandins that are produced. Rofecoxib, a selective COX2 inhibitor, worked well for adhesion prevention when given orally to mice [30]. Intramuscular indomethacin seemed more effective than oral Rofecoxib during testing on porcine pericardium. However, both have reduced prostaglandin E2 and thromboxane B2 generation [31]. Tenoxicam has not only exerted anti-adhesive properties after injection into the peritoneal cavity of rodents, but it has also lowered oxidative stress [32]. Intraperitoneal instillation of diclofenac in swine [33] and piroxicam in rodents [34] were also confirmed to be efficient. While both nonselective (naproxen, ibuprofen, indomethacin) and selective (Celecoxib, Rofecoxib) NSAIDs administered orally exhibit anti-adhesive properties, the latter appear to be superior. Celecoxib seems to have the strongest effect in the selective NSAIDs class [35].

Pentoxifylline, a methylxanthine derivative, injected either intraperitoneally or intravenously in mice, increased the tPA (tissue plasminogen activator) level and decreased the PAI (plasminogen activator inhibitor) level, which leads to fibrinolysis stimulation [36].

It is acknowledged that NSAIDs produce multiple side effects, such as peptic ulcer, gastrointestinal hemorrhage, nephrotoxicity and cardiovascular effects (myocardial infarction, congestive cardiac failure, hypertension, stroke) [37]. Taking that into consideration, local rather than systemic administration would probably be safer in adhesion prophylaxis.

➢ Ethanol

Ethanol was tested on swine with induced chronic cardiac ischemia. In comparison to the control group, which received a diet supplemented with sucrose, the animals who consumed alcohol developed fewer adhesions and reduced fibrosis and collagen deposition areas were observed in the myocardium, as well as a thinner pericardium. The myocardial expression of proteins associated with adhesion (including TGFβ1) was diminished, while matrix metalloproteinases (proteins which damage adhesion) expression was elevated [38].

➢ Small molecule inhibitors

QLT-0267 is an integrin-linked kinase (ILK) inhibitor, which decreased proinflammatory cytokine synthesis induced by fibrin in mesothelial cell cultures from mice. After inoculation in the peritoneal cavity of laboratory animals, adhesion severity and serum IL-6 declined [39]. This study suggests that fibrin’s role is more than just structural in adhesion formation since it also amplifies the inflammatory process.

Four small molecule compounds, called Rhosin, CK-666, Golgicide A and Bepridil have demonstrated their ability to reduce adhesion formation. The first three are actin modulators. Bepridil acts as a calcium channel blocker and possesses antianginal properties, but prolonged high dose administration discloses its arrhythmogenic potential. This indicates that calcium signaling pathways also participate in adhesion formation [40].

Pirfenidone, an antifibrotic and anti-inflammatory small molecule, also protects against oxidative stress. During an experiment on rodents, both oral and intraperitoneal administration of pirfenidone (but especially the latter) reduced TGFβ and IL-17 levels. As a result, fibroblast proliferation is inhibited and adhesion formation is prevented [41]. Unfortunately, this drug cannot be recommended for widescale use in humans because of its many side effects (elevated transaminases, rash and photosensitivity, nausea, diarrhea and fatigue) [42].

Trametinib is a MEK 1/2 inhibitor which is currently used to treat malignant melanoma. It acts by suppressing Erk 1/2 phosphorylation. This inhibits the conversion of mesothelial cells into myofibroblasts and prevents the generation of adhesions. The effect is proportional to the dose, but even in high doses trametinib is tolerated well and does not interfere with physiological tissue healing [43].

➢ Hormones

Estrogen’s anti-adhesive properties are debatable. On the one side, estrogen administration did not significantly prevent adhesions in patients with septate uteruses who received hysteroscopic metroplasty [44]. On the other hand, postoperative estrogen did prevent adhesion re-emergence in patients who required adhesiolysis. The mechanism probably lies in stimulating uterine wall reepithelization, which accelerates endometrial regeneration [45].

Ghrelin is a peptide hormone which is produced in the gastrointestinal tract, particularly in the stomach. When injected intraperitoneally in rodents, it blocks the TGFβ signaling pathway and acts as an antifibrotic and anti-inflammatory agent. It also diminishes collagen deposition and fibroblast to myofibroblast transition, thus preventing adhesions [46].

The anti-adhesion drugs aim to disrupt the molecular processes involved in adhesion formation, such as inflammation and fibrinogenesis, and may also provide a physical barrier to prevent tissue adherence. An effective anti-adhesion drug should possess certain important characteristics, including biocompatibility, biodegradability and non-toxicity to ensure patient safety. It should also have a long-lasting effect, minimizing the need for repeated administration, and be easily applicable during surgery. Furthermore, an ideal drug should demonstrate efficacy across various surgical procedures and be cost-effective. By addressing these essential features, anti-adhesion drugs have the potential to significantly reduce postoperative adherential syndrome and improve patient outcomes.

**Table 1:** A synthetic overview of the most relevant anti-adhesive mechanisms of drugs.

Drug	Anti-Adhesive Mechanism	Environment of Drug Testing/Use
AT1 receptor blockers AT2 agonists (C21) ACE inhibitors (Lisinopril)	Interference with the renin-angiotensin-aldosterone system reducing inflammation, fibrosis and extracellular matrix deposition [13,14,15,16]	Lisinopril–proved efficient in animal testing [16]
HIF inhibitors (YC-1) Antioxidants (NAC)	Fibrinolysis and angiogenesis inhibition Anti-inflammatory and fibrinolytic properties [17,18,19,20]	used on laboratory animals [18,19,20]
Statins an hypolipidemic drugs	Reducing fibrosis [21]	Proved in human patients [21]
NK-1R antagonists (CJ-12,255)	Fibrinolysis and oxidative stress reduction [22,23]	Used in laboratory rats [22]
Lubricin	Anti-adhesive glycoprotein [24]	Animal testing [24]
Chymase inhibitors (Suc-Val-Pro-Pheᴾ-(OPh)₂; TY-51184; NK3201)	Reducing fibrosis [25,26,27,28]	Used in hamsters [26]
Cromoglycate	Inhibiting mast cells [29]	Used in rabbits [29]
Anti-inflammatory drugs (Rofecoxib, Tenoxicam, Diclofenac, Naproxen, Pentoxifyline etc.)	Reducing inflammation by inhibiting COX enzymes [30,31,32,33,34,35]	Proved in animal testing [33,34]
Ethanol	Reduces fibrosis and collagen deposition, stimulates matrix-metalloproteinases [38]	Animal testing [38]
QLT-0267	Decreases proinflammatory cytokine synthesis [39]	Used in mice [39]
Rhosin, CK-666, Golgicide A	Actin modulators [40]	Animal tested [40,41,42]
Bepridil	Calcium channel blocker [40]	Animal tested [40,41,42]
Pirfenidone	Antifibrotic, inhibition of oxidative stress [41,42]	Animal tested [40,41,42]
Trametinib	Inhibits myofibroblasts [43]	Seen in human patients [43]
Ghrelin	Reducing fibrosis and collagen deposition, inhibits myofibroblasts [46]	Seen in human patients [46]

### 3.3. Inert Polymers

Adhesion barriers are widely used products in postsurgical adhesion prophylaxis. They do not interfere with the pathophysiological mechanism of adhesion formation, but they work by separating injured tissues from one another or from healthy tissues. Only Interceed, Seprafilm and Adept have been approved by the FDA [1], but other barriers will also be discussed in this chapter.

➢ Natural polymers

Based on hyaluronan

Hyaluronan (hyaluronic acid) is a glycosaminoglycan found in synovial fluid, vitreous bodies, blood vessel walls and also as a constituent of connective tissues. A cross-linked hyaluronan gel was applied at the end of surgical treatment of peritonitis in patients with small bowel perforations. Bowel obstruction incidence was considered low in these subjects, with the beneficial result being attributed to the gel [47].

In gynecological surgery, using cross-linked hyaluronan gel during myomectomy interventions has not only prevented adhesions. Women who have benefited from it also had a greater number of pregnancies in comparison with the control group. Apart from its barrier function, it appears that hyaluronic acid stimulates mesothelial cell multiplication in the peritoneum as well, thus supporting the postoperative healing process [48].

Hyaluronic acid possesses some valuable characteristics, which make it suitable as an anti-adhesive agent. Its molecule is hydrophilic and when mixed with water, tissues become hydrated. Hyaluronan has viscoelastic properties, it lubricates the surface it is applied on, it is antioxidant and anti-inflammatory and it promotes recovery [49].

Based on cellulose

Products Seprafilm and Interceed contain carboxymethyl cellulose.

Seprafilm is a membrane consisting of sodium hyaluronate and carboxymethyl cellulose. After one to two days following application, it turns into a gel which acts as a barrier between the layers of the serosa. It does not exert any pharmacological effect, is absorbed from the application site within a week and completely cleared from the body at 4 weeks after use by renal elimination. It was considered efficient and safe when administered to different anatomical regions in laboratory animals. Seprafilm’s effect was not influenced by contact with blood, ischemia or radiotherapy. It did not affect healing of the tissues, nor did it promote the development of sepsis or the extension of neoplasms. Moreover, it is not toxic, pyrogenic, mutagenic or an irritant. However, Seprafilm’s efficacy decreases in the presence of peritonitis [6].

In human patients, this barrier has been mostly used during intestinal surgery. Despite its success as an anti-adhesive agent, it must be mentioned that if Seprafilm is applied right on the anastomosis, a leak of luminal contents from the surgical connection is possible. The product has also been used in gynecological surgery with satisfactory results, although it is harder to handle during laparoscopy since it is fragile [50].

Interceed, launched in 1990, is actually the first resorbable membrane to be introduced on the market. It contains oxidized regenerated cellulose, which is resorbed from the administration site after one month. Unfortunately, the presence of blood at the site affects the product’s efficacy, so thorough hemostasis should be performed before using Interceed. In obstetrics and gynecology, the barrier prevents adhesion formation, leading to an increased number of pregnancies compared to control groups, without altering tissue regeneration. Interceed adjusts better to the outline of the organs than Seprafilm [2].

Apart from its barrier function, Interceed appears to elevate tPA expression in mesothelial cells, modifying the tPA/PAI-1 ratio towards fibrinolysis [51].

Based on chitosan

Chitosan is obtained after deacetylation of chitin, the principal component in the shell of arthropods [52].

Chitosan presents hemostatic and antibacterial properties, as well as the ability to inhibit TGFβ. The derivative N,O-carboxymethyl-chitosan (NOCC), resistant to blood and other biological fluids, has demonstrated relevant anti-adhesive action, without influencing the normal healing process [53].

Testing chitosan on mice prevented adhesions produced by trauma or ischemia but was ineffective against adhesions induced by foreign bodies. The reason lies in the generation of foreign body granulomas by talcum powder (which was used as the foreign body). The granulomas persist, but the chitosan gel is degraded in two weeks and will be unable to counteract the adhesion formation. Furthermore, combining chitosan and gelatin amplifies the adhesive process, probably because gelatin acts as an antigen and will be rejected by the body [52].

A study analyzed the effect that gels with different chitosan/gelatin ratios (100/0, 75/25, 50/50, 25/75) had on an experimental group of rodents, in contrast to the control group. Results showed that more than 25% chitosan in the product’s composition did not exert anti-adhesive properties and even stimulated inflammation and created more adhesions [54].

In conclusion, chitosan seems to be useful in adhesion prophylaxis. However, it should not be mixed with gelatin.

Natural membranes

REPEL-CV consists of polylactic acid and polyethylene glycol and has been approved in the USA for patients under 21 years of age who are receiving heart surgery and who will probably require a second cardiac intervention in the future. In addition to its barrier role involving separating the injured tissues from each other, REPEL-CV also acts as a framework for tissue healing [55].

This product has successfully reduced the severity and extent of adhesions in pediatric patients who received surgery for complex congenital heart defects [56].

SurgiWrap is based on polylactic acid. A retrospective case-control study which analyzed cases of laparoscopically treated colon neoplasms concluded that SurgiWrap decreased adhesions adjacent to the colostomies, thus facilitating colostomy closure [57].

➢ Membranes composed of synthetic polymers

Gore-Tex, containing expanded polytetrafluorethylene, was intensively researched in the 1990s. In 2003 a review was published assessing the numerous articles released prior to that time. The purpose was analyzing adhesion barrier efficacy in gynecological surgery and it showed that Gore-Tex was a better anti-adhesive agent than Interceed in laboratory animals. However, Gore-Tex has two major disadvantages: it requires suturing, which prolongs the intervention duration; and it is not absorbed from the application site, so it must be surgically removed. Nevertheless, even without surgical removal, this barrier did not cause any side effects when used as a vascular or pericardial graft and left in the body for a long period of time [58]. It was mentioned in two case reports that removing the barrier does not cause the formation of additional adhesions [59].

➢ Membranes containing collagen

In adhesion prophylaxis, collagen has the advantage that it acts as a base to which cells can attach and multiply, thus stimulating tissue regeneration. Collagen products have a good safety profile and are not difficult to manufacture.

Cova™CARD is a resorbable membrane consisting of purified type 1 collagen from swine. Its efficacy in cardiac surgery was compared with Preclude^®^ (non-resorbable membrane made of expanded polytetrafluoroethylene) and a control group by testing on sheep. Cova™CARD had already been completely absorbed at four months after initial surgery, when control sternotomy was performed. This experimental group exhibited reduced fibrosis areas and the highest incidence in epicardium repopulation with mesothelial cells, in contrast with the other two groups [60].

Cova™ has also been used during abdominal interventions in human subjects. As a result, patients displayed a significant reduction in adhesion number, severity and extension. The product was also easy to apply during laparoscopy and did not cause side effects [61].

TachoSil, a collagen sponge covered in fibrinogen and thrombin, is approved to be used for hemostasis. It also has anti-adhesive properties and was proved to be superior to Gore-Tex in pericardial adhesion prevention in laboratory rabbits. Additionally, TachoSil inhibits PAI-1 activity, tilting the balance in favor of fibrinolysis [62].

➢ Membranes made of mixed polymers

Prevadh is made of porcine collagen, polyethylene glycol and glycerol. It has been tested on rodents on which thoracotomy was performed and it prevented pleural adhesion formation altogether, without noticeable unwanted effects [63].

A randomized study has taken into consideration female patients whose uterine fibroids were excised during laparotomy, suggesting that Prevadh diminishes the incidence and severity of adhesions. At the same time, the chance of pregnancy after surgery increased. The following advantages of using Prevadh should be mentioned: satisfactory adherence at application site, persistence at the site for a few days, resistance to blood and complete degradation [64].

➢ Sprayable barriers

SprayShield, a polyethylene glycol barrier, can be sprayed on the areas prone to adhesion formation and is metabolized after one week. It includes two compounds, the polyethylene glycol solution and a buffer solution which contains blue dye. When the product comes in contact with tissues, it polymerizes and becomes a gel. Due to the blue color, the surgeon can easily check if the barrier has been applied to the right area. SprayShield has been tested on 11 patients suffering from ulcerative colitis or familial adenomatous polyposis. Although adhesion formation was less noticeable in the experimental group than in the control group, a statistically significant conclusion cannot be drawn since a small number of patients participated in the study [65].

A trial that included 15 patients undergoing myomectomy also had inconclusive results regarding the anti-adhesive properties of SprayShield [66].

To conclude, SprayShield should be tested on larger groups of subjects in order to establish whether or not it is effective in adhesion prophylaxis.

CoSeal^®^ Surgical Sealant consists of polyethylene glycol and is completely absorbed from the application site in one month. It was safe and useful in 71 women who required myomectomy, since the incidence of adhesions in the experimental group was three times lower than in the control group. No risk of intra-abdominal or surgical site infections was associated with this product [67].

CoSeal^®^ was also sprayed on the heart at the end of surgery for congenital cardiac defects in pediatric patients. Although there was no control group to compare results with, most adhesions that appeared in these patients were thin, translucent and contained few blood vessels. The barrier is relatively safe, since cardiac tamponade as an adverse effect was uncommon [68].

A similar study, which included both an experimental group and a control group, confirms that Adhibit, which has an identical composition to CoSeal, successfully prevents adhesions [69].

➢ Liquid barriers

Adept is a solution made of icodextrin 4%. Icodextrin, a glucose polymer, prolongs Adept’s absorption duration, so that it is cleared from the administration site slower than other crystalloid solutions. This explains why Adept is superior to Ringer’s lactate in adhesion prophylaxis. This product is absorbed in the lymphatic system in the first few days after the intervention and is then metabolized. The safety profile is satisfactory, except for the possibility of an allergic reaction [50].

Adept was proved to be safe and efficient when used during gynecological laparoscopic procedures which also included adhesiolysis [70].

➢ Membranes based on heterograft

There is little information available regarding heterograft membranes in the literature. One study analyzed the anti-adhesive effect of acellular pericardiums of bovine origin combined with hyaluronan in rabbits. Pericardial regeneration was demonstrated through immunohistochemistry and TNFα levels were lower in the treatment group than in the control group during the postoperative period. Bovine pericardium behaves like a barrier between the epicardium and the sternum, but also acts as a basis for tissue healing. Adding hyaluronan contributes to the adhesion prophylaxis [71].

### 3.4. Incorporation of Medical Substances in Barriers

It was previously discussed that anti-adhesive barriers physically separate tissues from each other, which means that no pharmacological effect is involved. Thus, the following hypothesis emerges: integration of pharmacologically active substances in barriers could increase the intensity of the anti-adhesive effect.

For example, tissue plasminogen activator (tPA) was incorporated in a gel barrier and tested on rodents. Scanning electron microscopy confirmed the repopulation of the injured area with mesothelial cells. A decreased PAI-1 level in the peritoneal lavage fluid has been revealed using ELISA [72].

A recent idea led to the creation of a lidocaine, carboxymethylcellulose and polyethylene oxide product. Lidocaine, a local anesthetic, also presents anti-adhesive properties. In-vitro testing shows that the new product is not toxic to cells. The level of cross-linking determines the rate of lidocaine delivery from the barrier towards the tissues [73].

Mitomycin, simultaneously an antibiotic and antineoplastic drug, induces DNA damage. In vitro fibroblast proliferation is suppressed An experiment was organized in order to decide whether or not the controlled release of mitomycin from a gel barrier could diminish adhesion generation. The results showed that gels containing both hyaluronan and mitomycin C were more efficient at adhesion prevention than gels without mitomycin C [74].

Another interesting research topic lies in the combination of anti-inflammatory drugs and barriers. It is assumed that arachidonic acid metabolites amplify adhesion formation through their proinflammatory action. Sustained ibuprofen release has considerably reduced the postoperative inflammatory response in laboratory animals, thus preventing adhesions [75].

### 3.5. Nanoparticles and Gene Therapy

Nanoparticles are particles with a size under 100 nanometers. They possess unique physicochemical properties, such as small dimension, high area-to-mass ratio and high reactivity. Advantages of nanoparticles are smart drug delivery (increased concentration of the medication in a specific area) and slow release of the drug in the body in a prolonged controlled fashion [76].

Silver ions are potent antibacterial agents but they are cytotoxic. Silver ions have been integrated in a poly(L- lactide) membrane by electrospinning. Inhibition of fibroblast proliferation, absence of cytotoxicity and antibacterial effect against Staphylococcus epidermidis, Staphylococcus aureus and Pseudomonas aeruginosa were noticed in vitro. Interestingly, the cytotoxicity of silver ions has been turned from a disadvantage to a valuable characteristic of nanoparticles since it especially affects fibroblasts that participate in adhesion generation [77].

Gene therapy can correct anomalies which appear immediately after surgery at a molecular level. It uses vectors which are administered locally at the end of the intervention. The alteration of gene expression is maintained for a short period of time, thus overlapping with the duration of molecular anomalies. This field is still in its initial stage of development, but it is considered promising [78].

At this moment, only a few studies regarding gene therapy in adhesion prophylaxis are found in the literature.

On cultures of fibroblasts which were sampled from human peritoneal adhesions, an adenovirus served as a vector that coded a human tPA gene (tissue plasminogen activator gene). The experiment succeeded since the adenovirus had successfully targeted fibroblasts with a role in adhesion formation and spared normal cells. As an idea for the future, a barrier which also contains this viral vector could be produced [79].

Adenoviruses have also delivered HGF (hepatocyte growth factor) genes [80] and SK-1 (sphingosine kinase 1) genes, which is a molecule involved in the same signaling pathway [81]. This has stimulated mesothelial cell multiplication and migration and has diminished adhesion formation.

It has been noted that bFGF (basic fibroblast growth factor) and VEGFA (vascular endothelial growth factor) genes are crucial for healing tendon injuries. Poly(lactic-co-glycolic acid) nanoparticles served as vectors that supplied tendons with the aforementioned genes. As a result, strength was greater in the group treated with one or both genes than in the control group and adhesion formation was reduced [82].

A fascinating discovery indicates that exosomes originating from human umbilical cord stem cells suppress the genes that lead to inflammation and fibrosis [83].

## 4. Conclusions

Postoperative adhesions are a very common issue in the healthcare system which cause considerable complications and an ideal prevention agent is yet to be designed. For these reasons, countless products have been analyzed and proposed for prophylaxis: from changes in the surgical technique to barriers and pharmacologically active compounds to new technologies (nanoparticles and gene therapy). Regarding the article’s strengths, we consider that it explains the mechanism of action and brings experimental evidence for each proposed product. As for limitations, the majority of the adhesion prevention options have only been tested on laboratory animals and are still far from being used in a clinical setting. Further research is required in order to decide which of all of these products is actually appropriate for patients.

In past years, substantial progress has been made in understanding the mechanisms which lead to postsurgical adhesion formation and a lot of products have demonstrated their efficacy in experimental studies. However, only a few of them are approved and are used widely at the moment. Unfortunately, in the clinical setting, adhesion prevention only consists of separation of the injured tissues from each other.

## Data Availability

All data that is cited can be found in the cited articles.
